# The pathway of left ventricular blood flow in healthy subjects and patients with corrected atrio-ventricular septum defect: an observational study using 4DFlow MRI and particle tracing

**DOI:** 10.1186/1532-429X-16-S1-P136

**Published:** 2014-01-16

**Authors:** Emmeline Calkoen, Patrick J de Koning, Arno Roest, Lucia J Kroft, Rob J van der Geest, Pieter J van den Boogaard, Monique R Jongbloed, Albert de Roos, Jos J Westenberg

**Affiliations:** 1 Pediatric cardiology, LUMC, Leiden, Netherlands; 2 Radiology, LUMC, Leiden, Netherlands; 3 Cardiology, LUMC, Leiden, Netherlands

## Background

Blood entering the left ventricle (LV) through the left atrio-ventricular valve (LAVV) and exiting through the aorta is assumed to follow the most energy efficient pathway. In patients with a corrected atrio-ventricular septal defect (AVSD), this intra-cardiac blood flow pathway may be altered, due to the restricted opening of the LAVV. This might affect optimal LV function. Particle tracing in 4DFlow MRI data enables quantitative evaluation of the temporal distribution of blood particles in the LV. We aimed to compare the LV intra-cardiac blood flow pathway in corrected AVSD patients with that of healthy subjects, using 4DFlow MRI and particle tracing in the standard 16 segment model of the American Heart Association (AHA).

## Methods

**Figure 1:**
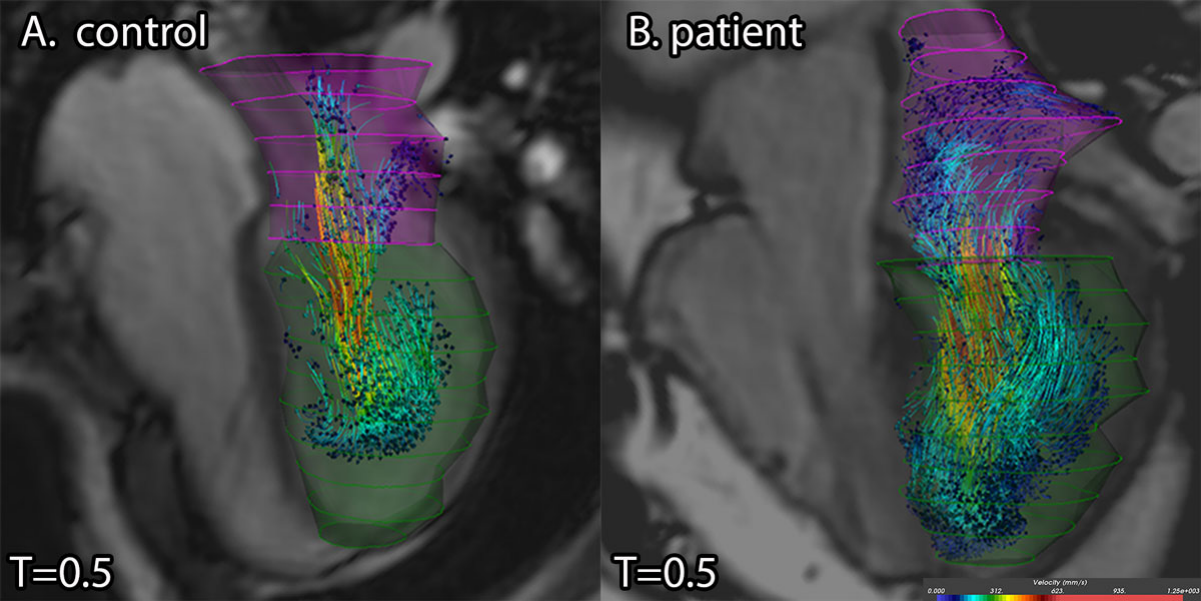
**An example of LV filling by using particle tracing, illustrating the difference between the blood flow pathway in a healthy subject (A) and a patient with corrected AVSD (B) at time point 0.5**. Colors indicate velocity (mm/s). Note the difference in apical filling.

Twenty five patients with a history of corrected AVSD (mean age 23 ± 10 years) and 25 healthy subjects (21 ± 11 years) were included. Whole-heart 4DFlow MRI was performed at 3T (Ingenia, Philips, The Netherlands) with free breathing, three-directional velocity encoding of 150 cm/s in all, spatial resolution 2.3 × 2.3 × 3.0-4.2 mm 3 and 30 phases reconstructed over one cardiac cycle. Particles, evenly distributed within the left atrium (LA), were released at the end-systole and then followed over one cardiac cycle using forward particle tracing (Figure 1). The pathway of these particles was assessed from the temporal particle distribution according to the standard AHA 16 segment model. The amount of particles per segment at a time point, is expressed in particle percentage (pp), as the fraction of total particles released at t = 0 (end systole) in the LA. Differences in pp within subjects were compared using paired t-test; differences between patient and controls were compared using unpaired t-tests.

## Results

**Figure 2:**
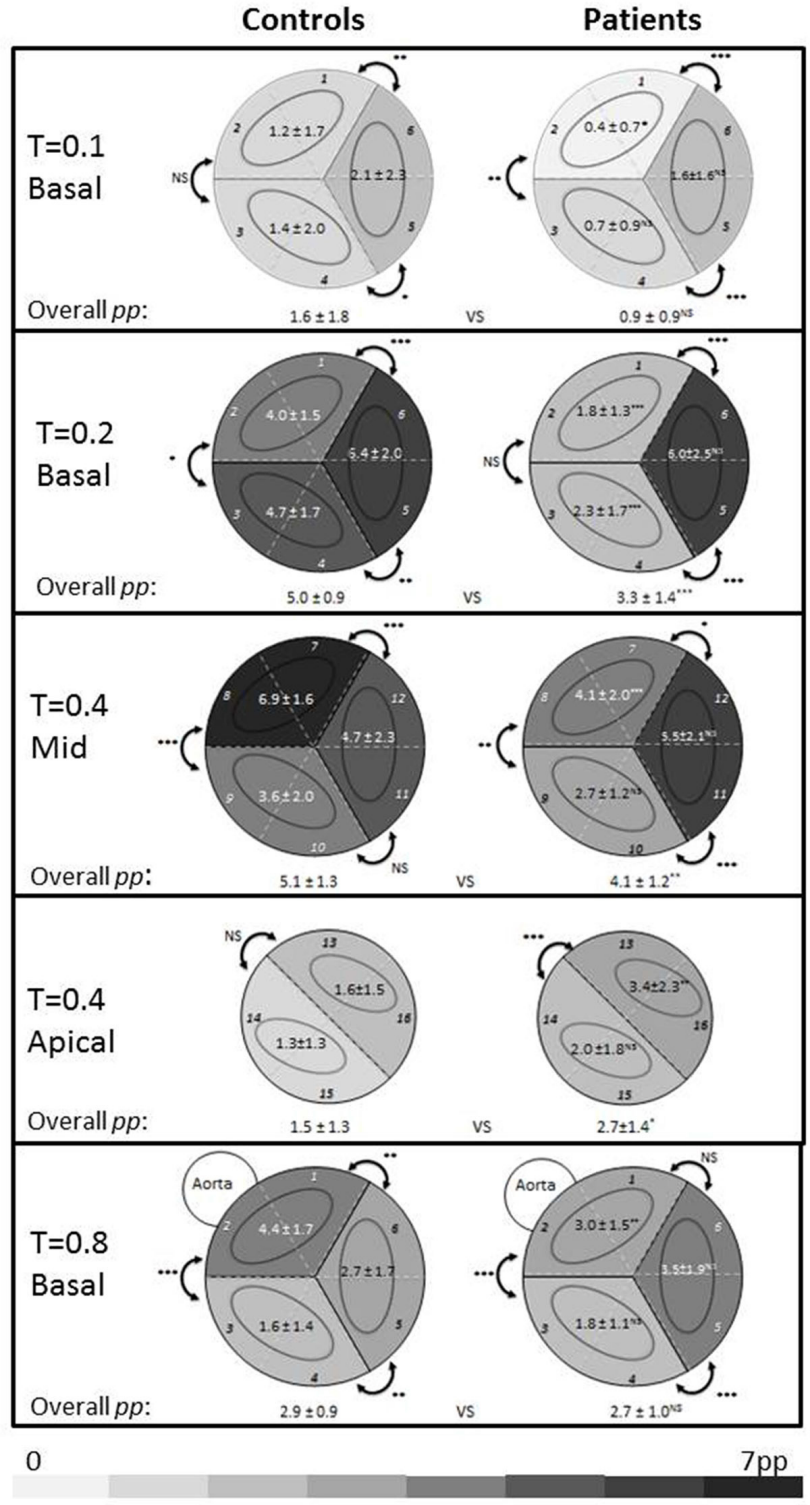
**Particle distribution (expressed in particle percentage (pp) as the fraction of total particles released) at four time points in the cardiac cycle at basal, mid and apical level, distributed according to standard 16-segment AHA model (1-16)**. Mean pp and standard deviation are given in the diagrams, for clustered neighbouring segments. Gray scale coding represents the amount of pp. Statistical significance between neighbouring segments and between patients and controls is expressed as follows: * = p < 0.05, ** = p < 0.01, ***p < 0.001. Segments: 1 Basal anterior, 2 Basal anterior septal, 3 Basal inferior septal, 4 Basal inferior, 5 Basal inferior lateral, 6 Basal anterior lateral, 7 Mid anterior, 8 Mid anterior septal, 9 Mid inferior septal, 10 Mid inferior, 11 Mid inferior lateral, 12 Mid anterior lateral, 13 Apical anterior, 14 Apical septal, 15 Apical inferior 16 Apical lateral.

In Figure 2, values for mean pp are presented for four typical time points. These diagrams reveal that firstly, in controls and patients, the start of early filling (t = 0.1) predominantly takes place laterally (p ≤ 0.04), followed (t = 0.2) by filling in all segments. In patients, filling through segments 1-4 is significantly lower (p < 0.001). At the end of early filling (t = 0.4), mean pp in controls is significantly higher (p < 0.001) in mid LV segments 7+8 (i.e. anterior). In patients, pp is significantly higher (p = 0.02) in segments 11+12 (i.e. lateral), and significantly more particles (p = 0.01) reach into the apical segments, especially anterior-lateral (p < 0.001). During systole (t = 0.8), particles move towards basal segments 1+2 towards the aorta, but in patients, mean pp in segments 5+6 (i.e., lateral) is equally high as particles in these segments may contribute to LAVV regurgitation.

## Conclusions

With 4DFlow MRI and particle tracing, an altered pathway in the intracardiac blood flow can be observed in patients with a corrected AVSD, that might affect optimal LV function.

## Funding

Willem Alexander Kinderfonds and Dutch Technology Foundation (STW) project number 11626.

